# Droplet-Based Measurements of DNA-Templated Nanoclusters—Towards Point-of-Care Applications

**DOI:** 10.3390/bios15070417

**Published:** 2025-07-01

**Authors:** Jonas Kluitmann, Stefano Di Fiore, Greta Nölke, Klaus Stefan Drese

**Affiliations:** 1 Institute of Sensor and Actuator Technology, Coburg University of Applied Sciences and Arts, Am Hofbräuhaus 1b, 96450 Coburg, Germany; 2 Fraunhofer Institute for Molecular Biology and Applied Ecology IME, Forckenbeckstr. 6, 52074 Aachen, Germany

**Keywords:** nanoclusters, microfluidics, fluorescence, segmented flow, Point-of-CareTesting, DNA-templated silver nanoclusters, biohybrid nanosensors

## Abstract

In this work, we investigate the fundamental usability of fluorescent DNA-templated silver nanoclusters (DNA-AgNCs) as sensors for Point-of Care-Testing (PoCT) applications. We developed a microfluidic platform for the generation of droplets containing DNA-AgNCs in defined, different chemical environments. The droplets are read out fluorescently at two different emission wavelengths. For the pre-evaluation for the usage of biologically relevant matrices with DNA-AgNCs, the response of two different DNA-AgNCs to a variation in pH and sodium chloride concentration was acquired. Our compact and simple setup can detect DNA-AgNCs well below 100 nM and allows the characterization of the fluorescence response of DNA-based biohybrid nanosensors to changes in the chemical environment within short measurement times. The model DNA-AgNCs remain fluorescent throughout the physiologically relevant chloride concentrations and up to 150 mM. Upon shifts in pH, the DNA-AgNCs showed a complex fluorescence intensity response. The model DNA-AgNCs differ strongly in their response characteristics to the applied changes in their environments. With our work, we show the feasibility of the use of DNA-AgNCs as sensors in a simple microfluidic setup that can be used as a building block for PoCT applications while highlighting challenges in their adaption for use with biologically relevant matrices.

## 1. Introduction

Point-of-Care Testing (PoCT) is a field of growing importance in diagnostic applications. It has turned into a cornerstone for near-patient testing or patient self-testing. PoCT has, among many other applications, improved HIV care by providing diagnostic markers in a timely manner for therapy adjustment [1], allowed for the rapid surveillance of infection-specific markers and contributed to infection control during the COVID-19 pandemic [2,3,4,5], down to the measurement of blood glucose levels by physicians and patients alike [6].

The definition of PoCT and requirements for systems qualified as such can vary depending on jurisdiction and guidelines. Common features of PoCT are a quick turnaround time, easy sample preparation and the simple usage of the system without in-depth training [7]. Setup and instrumentation as well as readout, however, vary significantly depending on the application: COVID-19 rapid antigen tests were usually realized as immunodiagnostic lateral flow strip assay, simple in construction and cheap to manufacture, utilizing capillary force as the liquid driver, necessitating only an easy one-step all-liquid-phase sample preparation and providing a readout by visual inspection [2,8]. At the other end of the complexity scale are cartridge systems that integrate multiple liquid handling steps for complex assay procedures, self-calibration procedures and electronically addressable readout sensors that require hardware for operation and readout [7,9]. For testing in primary care facilities, the WHO has established a set of criteria for an ideal PoCT system. The ASSURED criteria require a system that is Affordable, sensitive, specific, user-friendly, rapid and robust, equipment-free and deliverable to end users [10].

Analogous to the broad array of technical solutions of PoCT systems, there is also a large variety of sensors used. The sensors range from immunochemistry-, enzyme- and nucleic acid-based systems to electrochemical sensors and optical sensors using colorimetric, absorbance or fluorescence readouts for target detection.

Within this context, DNA-templated silver nanoclusters (DNA-AgNCs) are an intriguing class of optical biohybrid nanosensors with easy synthesis, robust photostability, a high quantum yield of up to 100% and unique fluorescence properties depending on the oligonucleotide sequence. Following changes in the chemical environment, DNA-AgNCs can exhibit changes in their fluorescence [11,12]. Advantageously, by varying the oligonucleotide sequence of the DNA template, the optical properties of the DNA-AgNCs can be tuned to emit fluorescence throughout the visible range well into the near-infrared range [13] with large stokes shifts [14] and universal excitation via the DNA bases at wavelengths in the range of 260–280 nm [15].

Many oligonucleotides yield multiple species of NCs in a synthesis. These can be dark, but fluorescent species that lead to superpositions in the fluorescence spectra can also exist [14,16]. However, single species with multiple emissions have been described [17,18]. DNA-AgNC species can be convertible from one to another by means of oxidation and reduction. Reversible conversions between multiple states, including dark states, have been described [19,20,21]. Thus, DNA-AgNCs can be regarded as dynamic systems, which is further substantiated by the exchange of silver atoms in the clusters, while the optical response of the collective DNA-AgNCs remains stable [22].

Due to these dynamic properties, it is not surprising that DNA-AgNCs can show specific responses to changes in their environment and have been used for many sensing applications. For example, the feasibility of turn-off sensors has been demonstrated for the detection of mercury compounds at low nanomolar concentrations [16,21,23,24]. Other examples include sensors for D-Penicillamine [25], tetracycline [26], a turn-on sensor for quinolones from Cu^2+^-deactivated DNA-AgNCs [27], potassium ions and nitric oxide [28] or tryptophan for sepsis detection [29]. Sensors for Point-of-Care applications must show a fast response time. The kinetics of the reactions leading to the fluorescence change upon environmental changes can demand reaction times ranging from minutes for a deactivation by Hg^2+^ [16] to 20 min for a D-Penicillamine sensor [25], but they can also take longer than an hour in the case of a turn-off sensor for tetracycline [26]. Hence, if high throughput is required, multiple analyses must be set up and conducted in parallel and read out after specified incubation times.

The use of silver-based nanoclusters as sensors in PoCT may, however, face a challenge: the presence of halides in the environment, including chloride, leads to structural and electronic changes that affect the optical properties, which often leads to a decline or quenching of the fluorescence of the DNA-AgNCs [26,30]. Hence, in the presence of chloride in physiologically relevant concentrations, e.g., approximately 100 mM for human blood plasma [31], DNA-AgNCs oftentimes have little remaining emission intensities. However, fluorescent DNA-AgNCs that have been synthesized without deliberate chloride addition have been shown to contain chloride residues [32] and display an enhancement in emission intensity at low millimolar chloride concentrations [30]. Furthermore, chloride can be integral part of fluorescent silver-based DNA-AgNCs, and some DNA-AgNCs have been reported to exhibit fluorescence at physiologically relevant chloride concentrations [32].

The use of PoCT systems requires an easy and automatable handling and processing of the assay process. For this purpose, microfluidics has proven to be a valuable tool. For our study, we chose a droplet-based approach. While droplet-based systems are technically more demanding because they require an additional immiscible phase and fluid actuator as well as well-defined surface wettability, droplet-based systems also provide notable benefits. With an appropriately chosen dispersing phase, the single droplets are separated from each other so that each droplet can be regarded as a well-confined environment with unique reaction conditions. Hence, generating multiple droplets under identical conditions allows for the multiple execution of assays. By varying conditions between the generation of multiple sets of droplets, sequences of different reaction sets can be generated in fast succession, allowing for a pseudo-multiplex measurement. In a segmented flow with slug-shaped droplets, the residence time distribution is almost ideally sharp. In this case, the immutable sequence of droplets allows each droplet to be identified by its position. The possibility of tracking single droplets enables the creation and subsequent measurement of droplets of varying conditions, e.g., concentration ramps, and access to combinatorics and the storage and retrieval of single droplets and sequences [33]. Additionally, droplets can be leveraged to circumvent the problem of mixing in microfluidics. The segment internal flow in moving droplets facilitates rapid mixing if radial flow components are introduced [34,35].

The use of the segmented flow principle should thus allow for a facile operation of the platform with the option for the storage of droplets in case long reaction times are required and multiple reactions are supposed to be run in parallel.

In this study, we demonstrate the usability of DNA-AgNCs as signal transducers in a droplet-based microfluidic system in different chemical environments and the potential for their use in PoCT applications.

## 2. Materials and Methods

For this work, two published DNA-AgNC constructs were used and synthesized according to the literature [36,37,38]. A detailed description of the synthetic procedure is described in the ESI.

The droplet generation chip was manufactured by laminating laser-structured thermoplastic sheets [39]. Lexan8010 polycarbonate sheets of 500 µm thickness (TC00110, Dr. Dietrich Müller GmbH, Ahlhorn, Germany) were structured by a CO_2_ laser cutter (V6.60, Universal Lasersystems, Scottsdale, AZ, USA), cleaned in isopropyl alcohol and water, dried at 120 °C in a vacuum for four hours, aligned and stacked between microscopy slides. The chip was obtained by the lamination of the stacks in a hot press (UVL 5 0, Maschinenfabrik Lauffer, Horb am Neckar, Germany) at a peak temperature of 147 °C with 4 min of holding time above 145 °C at 20 kPa pressure of the platens on the stacks in a vacuum.

The channel surfaces were hydrophobized in a procedure adapted from Jankowski et al. [40]. Briefly, 2% *v*/*v* of aminopropyl terminated polydimethylsiloxane (0.08–0.09% amine, viscosity 1800–2000 cSt, abcr, Karlsruhe, Germany) in silicone oil (M10, 10 cSt., Carl ROTH, Karlsruhe, Germany) was pipetted into the channels, and the channels were sealed with adhesive tape and incubated at 80 °C. After 90 min, the solution was exchanged for fresh solution to displace bubbles, and the incubation was continued for 90 min. This procedure was repeated until no more bubble formations were observed. Subsequently, the channel was flushed with isopropyl alcohol, dried and filled with 2% *v*/*v* 1,2-Epoxytetradecane (>=95% GC, TCI Chemicals, Tokyo, Japan), incubated for 90 min at 80 °C, flushed with isopropyl alcohol and dried.

**Figure 1:**
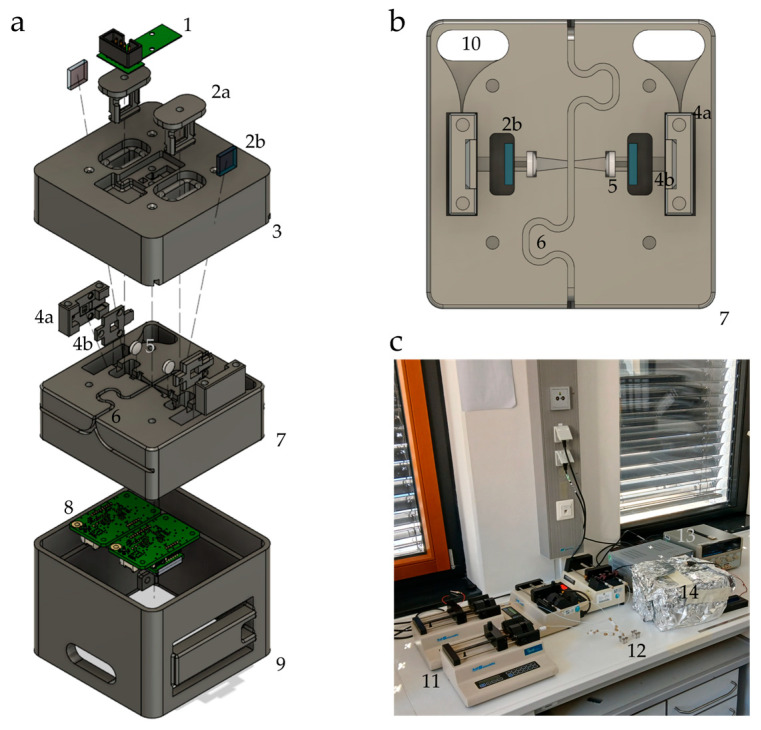
Schematics of the fluorescence readout cell and image of the laboratory setup. (**a**) Exploded view of the fluorescence flow-through measurement cell, (**b**) top-view of the optical setup in the measurement cell, (**c**) picture of the laboratory setup without computer for data logging. 1: emission LED board; 2a: filter holder; 2b: bandpass filter; 3: measurement cell slip lid; 4a: SiPM holder body; 4b: SiPM holder lid; 5: collimating lens; 6: trench for the tubing; 7: measurement cell body; 8: SiPM bias generator and amplifier module; 9: measurement cell electronics mount; 10: SiPM cable feedthrough; 11: syringe pumps; 12: droplet generator; 13: laboratory power supplies; 14: containment for the measurement cell. The LED constant current driver board and microcontroller board are not shown for clarity.

The fluorescence detection unit utilized an orthogonal layout. UV-C light from an LED (XST-3535-UV-A60-CE270-00, Luminus Devices Inc., Sunnyvale, CA, USA) driven at constant 85 mA was collected through a sapphire ball lens and restricted though a pinhole, irradiating a 1/16” outer diameter, 1/50” inner diameter PFA tube (48310-20, Microsolv, Greater Wilmington, NC, USA) with the segments passing through it. Orthogonal to the flow direction and irradiation, the emission was collimated through a lens (LA1116, Thorlabs Inc., Newton, NJ, USA), filtered through a bandpass filter (BP565-20K and BP668-15K, Tangsinuo, Shijiazhuang, China) and then detected by a silicon photomultiplier (SiPM) (C13367-3050EA, Hamamatsu KK, Hamamatsu Photonics K.K., Hamamatsu City, Japan). The analog voltage output of the SiPM module was captured using a TI ADS1256 module board (Hailege Technology, Shenzhen, China) at 120 SPS and recorded by a computer via an ESP32 microcontroller unit. The fluorescence detection unit was modeled with a CAD program and 3D printed in PLA. Figure 1 shows an exploded view of the construction and a top view of the optical path.

The authors feel compelled to point out that UV-C radiation is invisible and dangerous to eyes and skin. Proper precautions must be taken and evaluated prior to the testing and operation of the illumination unit.

The setup was driven by syringe pumps (KDS Model 200, Harvard Apparatus, Holliston, MA, USA) equipped with glass syringes of appropriate sizes (Gastight Series 1000 and Series 1700, Hamilton, Reno, NV, USA). The channel volume from the droplet generation site to the detection volume was 130 µL, equivalent to 112 s residence time of the droplets. A constant flow of 30 µL/min silicone oil with 10 cSt viscosity was applied, into which an aqueous phase with the DNA-AgNC system was dosed at 40 µL/min. Droplets of different compositions were generated by varying the flow rate ratios of the aqueous solutions.

## 3. Results

### 3.1. System Performance

**Figure 2:**
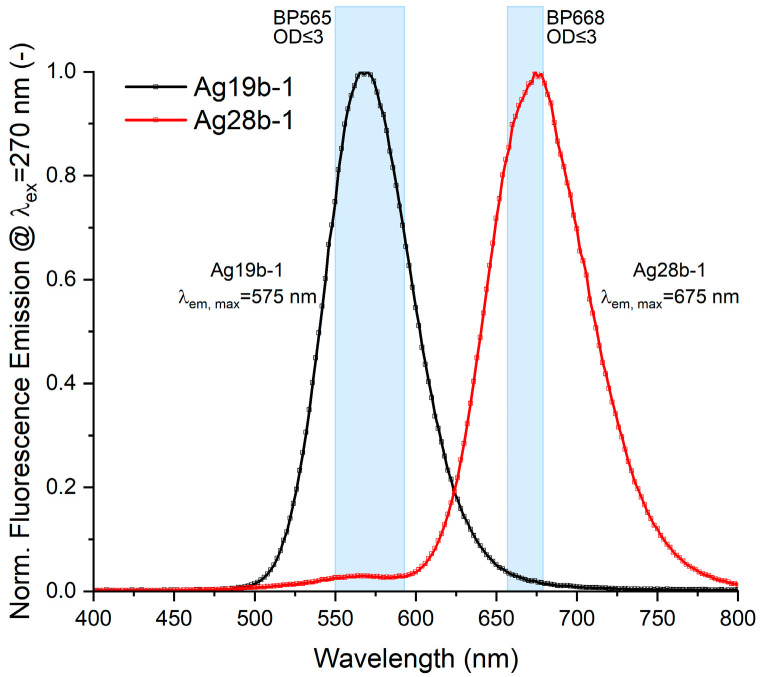
Normalized fluorescence emission spectra of the DNA-AgNCs in 20 mM NH_4_OAc. Excitation wavelength λ_ex_ = 270 nm. The shaded ranges show the transmission windows of the bandpass filters at ODs ≤ 3 used in the setup.

For the two synthesized DNA-AgNCs, the emission spectra for excitation at λ = 270 nm were acquired (Figure 2). Construct Ag19b-1 shows a single peak emission at λ_em,peak_ = 575 nm, while Ag28b-1 has one dominant emission peak at λ_em,peak_ = 675 nm and a second, minor emission peak at λ_em,peak_ = 565 nm that forms a shoulder on the blue side of the spectrum. The occurrence of two peaks or asymmetric peaks has often been connected to the presence of multiple emissive species of DNA-AgNCs [14].

Bandpass filters BP565 and BP668 were chosen to transmit around the emission maxima of the respective DNA-AgNC construct while blocking the emission of the other construct. Construct Ag19b-1 shows a small overlap of the red region of its emission spectrum with the transmission of filter BP668 which was chosen to transmit the emission peak of construct Ag28b-1. Conversely, the secondary emission peak of the Ag28b-1 construct is in the same region as the main emission peak of Ag19b-1, causing emission crosstalk. Hence, the emissions of both constructs were expected to be measurable in both channels, but with the emission of Ag19b-1 mainly measured in the BP565 channel and the emission of Ag28b-1 mainly measured in the BP668 channel.

To test the setup and the cross-sensitivity of the channels, dilutions ranging from 5 µM to 12.5 nM of the two model DNA-AgNC constructs were prepared in 20 mM ammonium acetate at pH 7, using an automated microfluidic serial dilution process. To cover this range, two syringes containing either 5 µM or 0.5 µM of the DNA-AgNC and one syringe with the diluent were used to mix the sample and diluent at flow rate ratios and conditions as indicated in Table S2.

**Figure 3:**
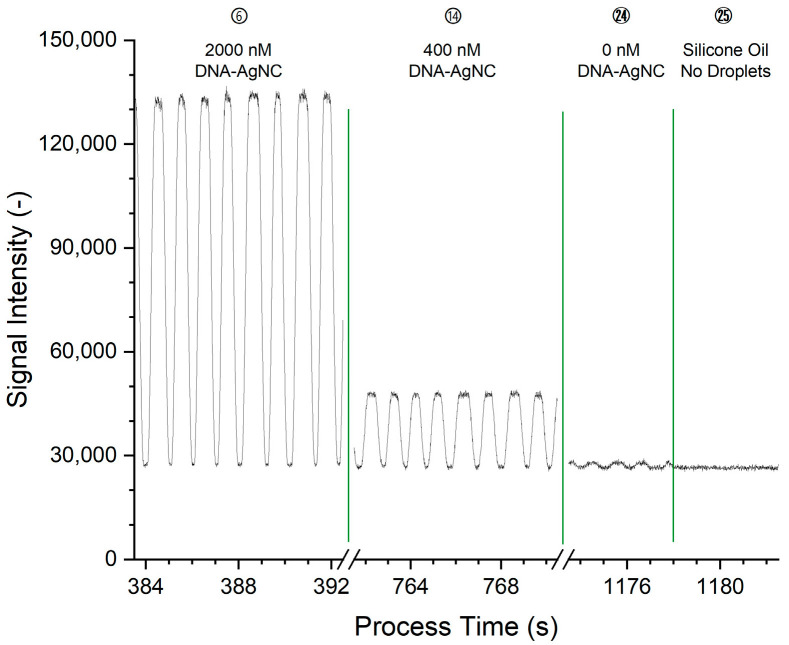
The raw data of the SiPM intensity signal for the dilution of the DNA-AgNC construct Ag28b-1 for channel BP668. The encircled numbers correspond to the condition number and match those in Figure S2 and Table S2.

Excerpts of the raw data for the measurement of construct Ag28b-1 through its main emission channel BP668 are shown in Figure 3. The corresponding processing of the data is shown and detailed in Figure S2.

The plot of the raw data shows peaks that correspond to the passage of DNA-AgNC-laden droplets through the detection volume, at a rate of approximately 1/s with pronounced plateaus. Since elongated slugs were generated, the shape of the peaks can be explained as follows: (I) the curved front surface of the slug moves into and through the detection volume, leading to the rising edge of the peak; (II) this is followed by the occupation of the detection volume by the main part of the slug, leading to the plateau of the peak; (III) as the slug moves out of the detection volume, the falling fraction of the detection volume occupied by the fluorescing slug and the curved surface of the slug end lead to the falling edge of the peak. Once the slug has left the detection volume, the baseline of the non-fluorescent PDMS carrier can be seen. The plateau intensity is constant and shows no fronting or tailing which would indicate an accumulation of the fluorophore at either part of the droplet at the given conditions. At higher fluorophore concentrations as in the early conditions, the signal intensity is higher. The raw signal in Figure 3 for condition 24 shows that peaks are also present for droplets that do not contain fluorophores. These peaks are ascribed to the difference in refractive index between the silicone oil and the aqueous phase, leading to different amounts of light scattered in the direction of and through the bandpass filters. The raw data was processed and averaged to one measurement value per condition, equivalent to one concentration step, as detailed in Figure S2.

**Figure 4:**
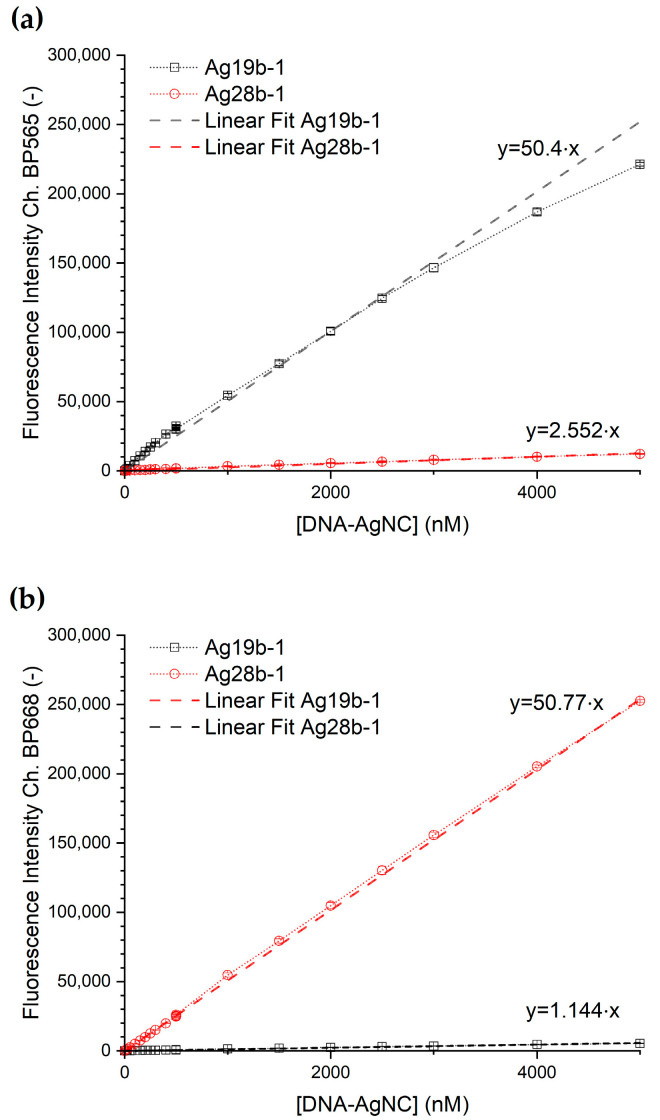
The concentration-dependent emission signal intensity of the DNA-AgNCs and linear fits of the datasets. (**a**) The signal of channel BP565 and (**b**) signal of channel BP668 for a dilution range of 5 µM to 12.5 nM for Ag19b-1 and Ag28b-1, respectively.

The concentration-dependent emission intensity of the DNA-AgNC per each detection channel is shown in Figure 4. The corresponding transfer functions from concentration to fluorescence intensity were obtained via a linear fit of the data for each DNA-AgNC construct for each channel with a fixed y-axis intercept at y = 0. Both dilution series show for both channels a rise in fluorescence intensity with a rise in DNA-AgNC concentration. Construct Ag28b-1 shows, furthermore, a linear response in the concentration range up to 5 µM, while construct Ag19b-1 shows a section-wise linear response with a break between the two linear ranges at a concentration of 500 nM in the BP565 channel. The corresponding data is shown in detail in Figure S3. To simplify further data analysis, the dataset was treated like one linear relationship.

The fitting parameters for the transfer functions following the linear relation (1) are shown in Table 1.

y = ax + b,(1)

The baseline noise of the measurement as the standard deviation of the signal intensity was determined for flowing silicone oil without droplets over a 10 s interval (Figure 3, starting at 1178 s). For the respective main emission channels, the baseline noise is 697 AU for Ag19b-1 in channel BP565 and 421 AU for Ag28b-1 in channel BP668. For Ag19b-1, the limit of detection (LOD) is hence 41.5 nM, and the limit of quantification (LOQ) is 138.3 nM. For Ag28b-1, the LOD is 24.9 nM, and the LOQ 82.9 nM.

### 3.2. The Response of the DNA-AgNCs to Chloride

To demonstrate the potential of the DNA-AgNCs for sensing in a droplet-based microfluidic system, the fluorescence responses of the DNA-AgNCs towards chloride ions were investigated. For both DNA-NC constructs, the signal intensity for chloride concentrations between 0 mM and 150 mM was acquired at a constant DNA-AgNC concentration in the droplets of 1.25 µM. To adjust the chloride concentration, solutions of (1) 20 mM ammonium acetate and (2) 200 mM sodium chloride and 20 mM ammonium acetate solution were mixed at different ratios during droplet generation.

**Figure 5:**
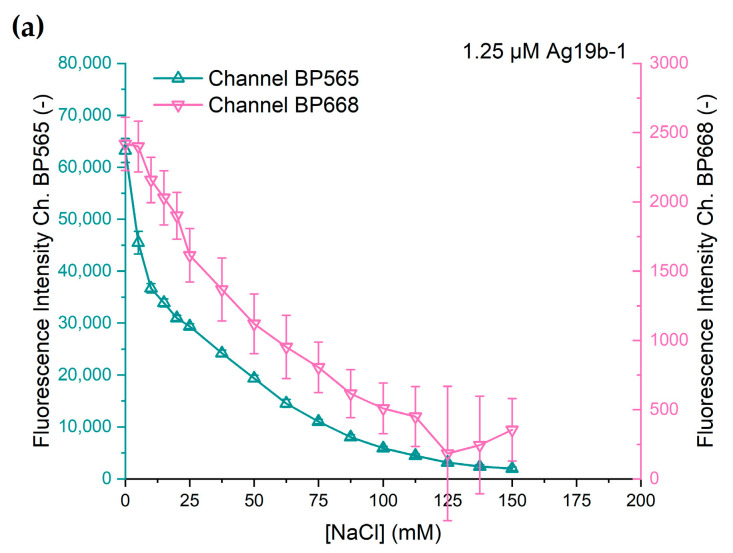

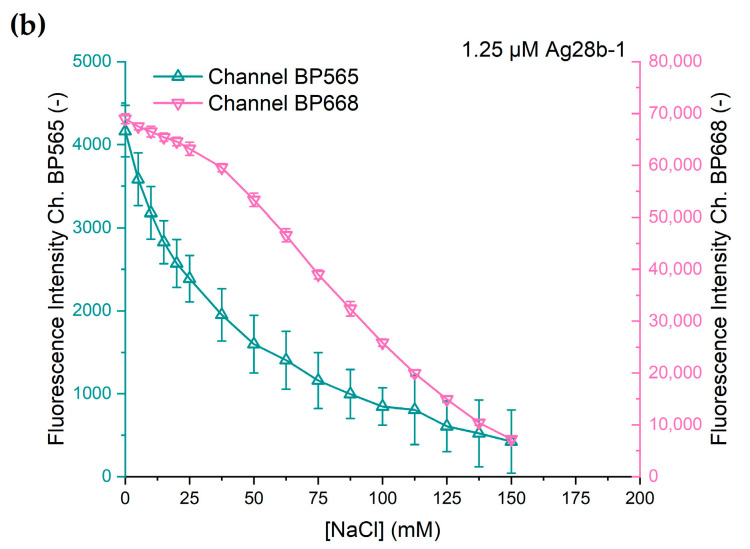
Fluorescence intensity of (**a**) Ag19b-1 and (**b**) Ag28b-1 in response to different concentrations of sodium chloride. Each datapoint consists of 33 ± 3 droplets, each representing one measurement. Error bars indicate the standard deviation.

The change in fluorescence intensity at different chloride concentrations of both DNA-AgNC constructs is shown in Figure 5. Both DNA-AgNC constructs show a decline in fluorescence intensity with rising sodium chloride concentration. However, the characteristics of the decline in fluorescence intensity vary between the constructs and the channels.

Ag19b-1 shows an initially strong decline in intensity at low concentrations of chloride with a slower decline at further rising concentrations and the fluorescence intensity asymptotically approaching zero at chloride concentrations above 150 mM. The fluorescence intensities of both channels show similar characteristics. This is an expected outcome since Ag19b-1 shows only one fluorescence emission peak and likely consists of one emissive species. Therefore, the deactivation of the emissive species is expected to quench the entire emission spectrum and thus in the BP565 channel and in the BP668 channel at the same ratio.

The response of Ag28b-1 is different for the two fluorescence channels. While the characteristic for channel BP565 is similar to that of Ag19b-1, the fluorescence intensity captured through channel BP668 shows a different response. Up to a chloride concentration of 25 mM, the decline in fluorescence intensity is small and quasi-linear. Starting between 25 and 37.5 mM, the decline in fluorescence intensity is stronger but also quasi-linear. At 150 mM chloride concentration, the fluorescence intensity decline slows down, probably also towards asymptotic behavior as exhibited with the other fluorescence responses. The difference in the responses from the two channels can be explained as the response of two different emissive species, each predominantly measurable in their own channel.

The comparatively large error bars can be attributed to the difference in the scaling of the channels. Compared to Figure 4, the scaling for the secondary channels in Figure 5 means a 100- or 60-fold magnification in the y-axis. The error data is shown in more detail in Figure S4.

### 3.3. Response of DNA-AgNCs to pH Changes

In a second set of experiments, we investigated the effect of pH shifts on the DNA-AgNCs. Analogous to the effect of chloride, the fluorescence of the DNA-AgNCs was quenched by adding acetate buffer to the DNA-AgNC solution. A final DNA-AgNC concentration of 1.25 µM and 5 mM ammonium acetate in the droplets was used. An acetate buffer of different pH values was obtained at the droplet generation by mixing solutions of (1) 40 mM acetic acid and (2) 40 mM sodium acetate at appropriate ratios.

**Figure 6:**
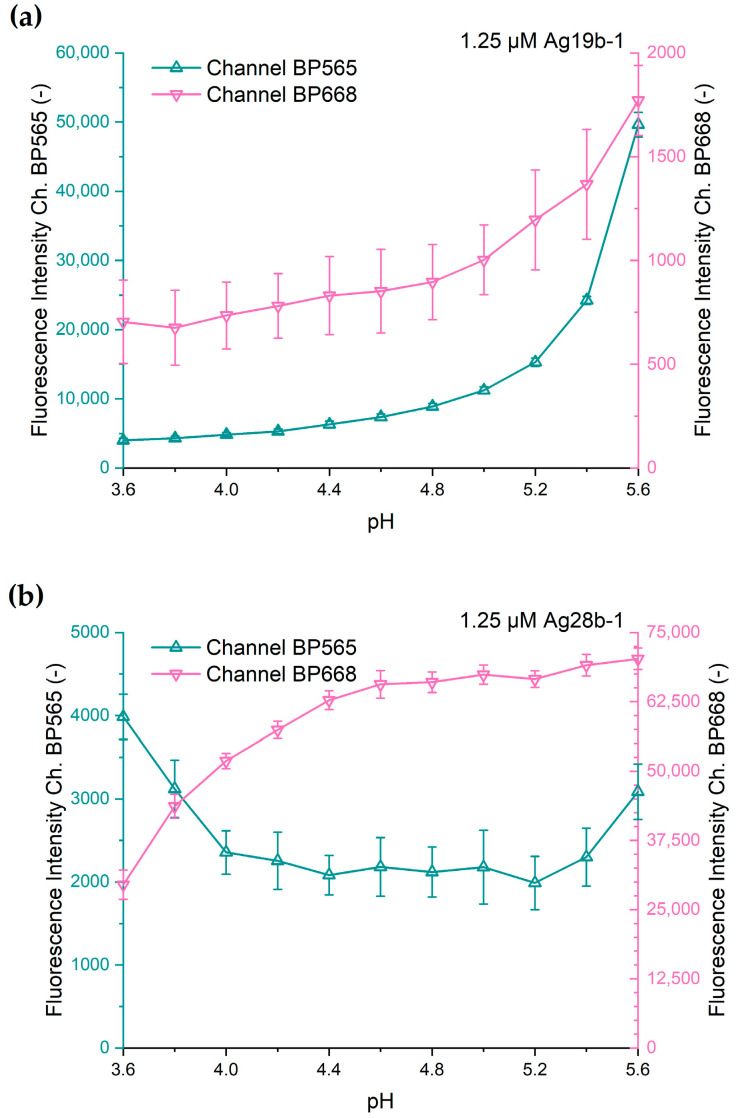
Fluorescence intensity of (**a**) Ag19b-1 and (**b**) Ag28b-1 in response to different pH values. Each datapoint consists of 33 ± 3 droplets, each representing one measurement. Error bars indicate the standard deviation.

Both DNA-AgNC constructs show fundamentally different fluorescence intensity changes at acidic pH values (Figure 6).

Ag19b-1 shows, for both channels, an initially strong decline in intensity at pH values below 5.6, with a slowing decline at progressively lower pH values. In the surveyed pH range, the full quenching of the fluorescence intensity was not observed. The fluorescence emission for channel BP668 shows similar results, but with a weaker decline in fluorescence intensity compared to channel BP565. However, the precision of the measurement was low due to the low signal intensity in the BP668 channel. The strong initial decline suggests that the emission optimum for Ag19b-1 lies at a considerably more basic pH than 5.6.

Interestingly, construct Ag28b-1 shows behavior that is strongly different to that of Ag19b-1 as the effect of the pH differs distinctively for both fluorescence channels. For channel BP668, the fluorescence intensity remains almost constant in the range of pH 5.6 to 4.6. At lower pH values, the decline in fluorescence intensity is progressively stronger. The signal intensity in channel BP565 shows a drop from pH 5.6 to 5.2, remains nearly constant until pH 4.2, and increases again at values down to pH 3.6. As for the chloride-induced changes in fluorescence, the differences in the responses for the different channels can be explained with the presence of two different species of DNA-AgNCs. The rise in intensity in the BP565 channel at the acidic end of the pH range is not consistent with the hypothesis of a simple deactivation, in which case an increase in signal intensity would not be expected. Instead, we speculate that (I) either a new DNA-AgNC species is formed that shows an emission in the BP565 channel or (II) the dominant species, formerly emitting in the range of the BP668 channel, exhibits a progressive blueshifted emission at a relatively constant emission intensity at falling pH values. A blueshift would initially not lead to a strong decline in intensity in the BP668 channel until the emission peak moves progressively out of the detection window of the bandpass filter. This could explain the initial constant slow drop in fluorescence intensity in the BP668 channel, followed by progressively stronger intensity declines at lower pH values. Conversely, the progressive shift of the emission of the dominant species into the BP565 channel could counteract the intensity decline of the secondary species. This could lead to constant intensity in this channel until the progressively stronger signal in the channel from the dominant species leads to an intensity rise in this channel again. These hypotheses cannot conclusively be investigated with our setup and require the acquisition of spectrally resolved data. Nevertheless, it must be pointed out that the lower signal intensities for the measurements in the BP565 channel produce lower precision, as indicated by the larger error bars.

The striking difference in response to the pH demonstrates that DNA-AgNCs can effectively and specifically respond to changes in the chemical environment.

A comparison of the data of the emission intensities of the dilution measurements of the DNA-AgNCs with those in the concentration response measurement of chloride clearly shows that the emission intensities for the concentration of 0 mM sodium chloride in Figure 5 do not match the concentration calibration data shown in Figure 4. The reasons for this behavior could include inconsistencies in the measurement solutions, a degradation of the AgNC sensor or slight changes in the beam path or light excitation intensities between the multiple experiments. Hence, a calibration of the zero-emission intensity and the intensity of the AgNC sensor should be carried out at each measurement.

One option to improve reproducibility would be the use of ratiometric measurements, which would lower the dependence of the measurements on the absolute fluorescence intensity.

**Figure 7:**
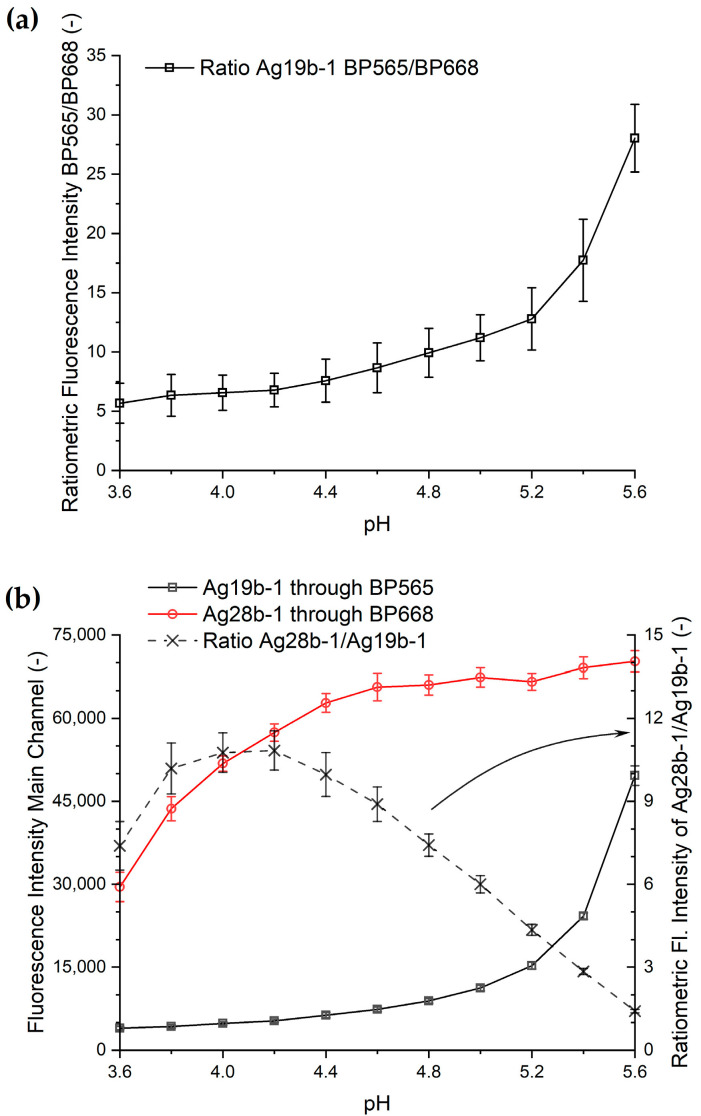
Proposed sensors for ratiometric pH measurements: (**a**) the use of Ag19b-1 in channels BP565 and BP668 and (**b**) the use of Ag19b-1 in channel BP565 and Ag28b-1 in channel BP668. The values of the dashed line are measured by the secondary y-axis of the chart as indicated by the arrow.

Based on the data presented in this study, we propose two options to construct a ratiometric pH sensor. The corresponding ratiometric data derived from our model measurements is shown in Figure 7. For the analysis of changes in pH, the constant intensity of Ag28b-1 in the BP668 channel renders this construct suitable as an internal standard for ratiometric measurements for pH values in the range of 5.6 to 4.6. The strong decline in the intensity of Ag19b-1 in the BP565 channel makes it suitable as a variable component in a ratiometric sensor. The difference in the decline in the ratiometric intensity of Ag19b-1 in the two channels could also enable it to work as a ratiometric sensor itself. The weak signal in the BP668 channel for construct Ag19b-1, however, leads to large uncertainties in the derived ratiometric measurement.

The use of DNA-AgNC species with only one emission peak as a ratiometric sensor implies that this species must progressively change their fluorescence properties by, e.g., shifting the emission maximum to another wavelength upon changes in pH or ion concentration. This would allow one to measure changes in fluorescence intensity at two emission wavelengths for the same sensor and use the ratio of these two emission values to sense a molecule or a change in the environment surrounding the sensor. Our setup does not, however, provide sufficient data to discriminate between the mechanisms leading to these effects due to the lack of more detailed spectral information. The use of two different constructs also bears the problem of the cross-sensitivity of the constructs outside of their main emission channel, as shown in Figure 3, further complicated by the nonlinear effect strengths of the effectors, as shown in this section.

## 4. Discussion

The results of our measurements show that the surveyed DNA-AgNCs have the potential to be used as sensors for biological sample matrices; however, the presence of chloride can pose a significant problem for the use of silver-based DNA-NCs. For the two sampled DNA-AgNCs the data shown in Figure 5 shows a strong dependence of fluorescence intensity on the chloride concentration over the entire sampled range with no obvious concentration threshold for non-interference. The problem pertains to a lowered emission intensity and to the uncertainty of the quenching efficiency due to uncertainties in the chloride concentration.

The range of chloride in different human biological sample types under normal conditions is <50 mM for sweat, 90–110 mM for blood [41], 115–130 mM for cerebrospinal fluid [42], 10–30 mM for saliva [43,44], and an amount of 135 mmol per day in urine [45]. Urinary chloride is particularly expected to pose a significant challenge as its concentration is dependent on fluid intake status [46]. The use of DNA-AgNCs for measurements of biological samples thus requires either (I) sample preparation to remove chloride and its interference with the measurement or normalize it to a known concentration, (II) an independent measurement of chloride to correct for its interference, (III) a dependent measurement, e.g., of a further DNA-AgNC, to correct for the chloride influence in a ratiometric measurement or by assessing spectral emission shifts or (IV) the dilution of the sample to a point at which the chloride concentration has negligible influence on the measurement result.

For the pH, our results indicate that DNA-AgNCs can exhibit a constant emission intensity over a pH range. This is in accordance with other investigations of DNA-AgNCs that also find pH ranges over which the emission intensity is not affected [25,47]; there are, however, also investigations that describe DNA-AgNCs exhibiting a sharp peak in the correlation between pH and emission intensity, albeit measured at pH steps of 1 between measurements [48,49,50]. This pH range is, however, individual to a specific DNA-AgNC and must be evaluated to be applicable to the pH of the target sample type. Corrections as proposed for the chloride interference are possible, while shifting the sample pH with a suitable buffer towards a value at which the DNA-AgNC emission exhibits no dependence on small pH shifts is an attractive option for sample preparation.

In addition to the chloride concentration and pH, free thiols are especially expected to interfere with DNA-AgNCs [25,47,51].

The use of DNA-AgNCs with biological sample matrices has been previously reported. The preferred method to mitigate the influence of the matrix in a strong dilution of the samples: The quantification of quinolones spiked into samples of urine has been conducted at a urine dilution of 1:100, which was the final concentration. The same study found that the DNA-AgNC construct was stable for more than 30 min in urine diluted 1:1000 [27]. For the measurement of histidine from a spiked urine sample, the urine was diluted 1:100 and the measurement solution treated with N-ethylmaleimide to prevent interference from thiol groups [48]. The detection of spiked D-penicillamine in serum has been conducted with serum diluted 1:1000, while proteins were precipitated using acetonitrile [25]. For the detection of bleomycin in serum, the serum was depleted from protein with acetonitrile and diluted 1:2000 in the measurement [52]. The detection of ATP and ADA in fetal bovine serum (FBS) was conducted with 1:100 pre-diluted FBS [50].

The integration of strong dilution steps into fluidic structures requires the use of sophisticated dilutors, e.g., droplet metering-and-merging systems [53]. While systems for this purpose exist, they are specialized constructs tailored and oftentimes hard-wired to their specific application. The transfer of the dilution strategy for analyzing biological matrices using DNA-AgNCs is hence challenging. The analysis of biological matrices that require dilution should be based on pre-dilution and pre-treatment to remove interfering components outside of the microfluidic device presented in this study. The diluted samples can be then mixed into the DNA-AgNCs and further analyzed in droplets as described above.

## 5. Conclusions

We presented a compact, easy-to-build and -control system based on a fluorescence readout that can be microfluidically addressed for measurements using DNA-AgNCs. Our approach shows for the first time that DNA-AgNCs confined into droplets are suitable to achieve the rapid measurement of change in pH or sodium chloride concentrations in solution. This is the first step towards the exploration of the direct applicability of DNA-AgNCs with biological matrices in a microfluidic environment. In further developments, a measurement from a biological matrix and measurements for analytically relevant endpoints will be implemented based on this setup. The large variety of published sequences of DNA-NCs, with relevance to the analysis of a broad analyte spectrum, opens up multiple possibilities to explore the capabilities of our detection platform. The fast analysis times are a beneficial factor for PoCT applications as well Point-of-Use applications for process analytics. Our droplet-based system is not equipment-free and thus does not meet all of the WHO′s ASSURED criteria. However, we are convinced that by improving some features in the setup of the system such as its handling and portability, we can unfold the potential of DNA-AgNCs by harnessing the features of this intriguing and versatile class of nanosensors for microfluidics-based PoCT applications.

## Figures and Tables

**Table 1:** Fitted parameters for the linear transfer function from concentration to fluorescence intensity. Values are given with standard error. For all fits, all 11 concentration points were used.

Dataset	a	b	R^2^
Ag19b-1 BP565	50.4 ± 1.2	0	0.9721
Ag19b-1 BP668	1.144 ± 0.019	0	0.9940
Ag28b-1 BP565	2.552 ± 0.044	0	0.9937
Ag28b-1 BP668	50.77 ± 0.16	0	0.9998

## Data Availability

The original contributions presented in this study are included in the article/Supplementary Materials. Further inquiries can be directed to the corresponding author.

## Supplementary Materials

The supporting information can be downloaded at https://www.mdpi.com/article/10.3390/bios15070417/s1, Table S1. DNA sequences of the oligonucleotides used for the synthesis of DNA-AgNCs in this study; Figure S1. Spectra of the bandpass filters used in the setup. (a) absorbance spectrum and (b) transmission spectrum; Table S2. Flow rates, corresponding concentrations and condition number corresponding to Figure 3 and Figure S2; Figure S2. Data processing steps from the raw datatrace to the final emission intensities used for the data analysis. (a) Raw datatrace of the ADC output for channel BP668 for the dilution experiment of Ag28b1. The green, vertical lines denote changes in flow rate ratios and consequently different compositions of the droplets. (b) Detail of the raw data for condition 15, shaded areas are the truncation of the leading and trailing droplets that have been excluded from further analysis. (c) 20 pt. moving average applied to the raw data and marked de-tected peaks for the process time interval marked in the previous panel. (d) Plot of the height of the detected peaks over the process time. (e) Plot of the mean and standard deviation of the peak heights for every condition and offset of the data to correct for the intensity of non-fluorescing droplets. (f) Detail of the last flow conditions at 12.5 nM DNA-AgNC, 0 nM DNA-AgNC and silicone oil without droplets, respectively; Figure S3. Fluorescence intensity over the concentration of Ag19b1. With linear fits for the ranges 0 500 nM and 500 2500 nM.; Table S3. Fitted parameter for the section-wise linear transfer function from concentration to fluorescence intensity for Ag19b1 for the BP668 channel.; Figure S4. Error of the fluorescence intensity (a) over the concentration sodium chloride for all deactivation experiments and (b) over the fluorescence intensity.

## Abbreviations

The following abbreviations are used in this manuscript:

BP	Bandpass
DNA-AgNCs	DNA-Templated Silver Nanoclusters
LOD	Limit of Detection
LOQ	Limit of Quantification
PoCT	Point-of-Care Testing
PDMS	Polydimethylsiloxane
SiPM	Silicon Photomultiplier
