# Supplementary material for: Droplet-Based Measurements of DNA-Templated Nanoclusters—Towards Point-of-Care Applications
Source: Biosensors (Basel). 2025 Jul 1;15(7):417. doi: 10.3390/bios15070417 (PMC12294068; doi:10.3390/bios15070417)
Supplement: Supplementary file 1 [file biosensors-15-00417-s001.zip › biosensors-3553864-supplementary.docx]

**Synthesis of the DNA-AgNCs**

The DNA-AgNCs were synthesized according to literature [1]. Oligonucleotides were purchased from IDT (Standard desalted, Leuven, Belgium). Silver nitrate (AgNO_3_, ≥99.998 %), sodium borohydride (NaBH_4_, 99.99 %) and ammonium acetate (NH_4_OAc, 99.99 %) were purchased from Sigma Aldrich and used as received. All solutions were prepared in double distilled water. DNA-AgNCs were synthesized as follows: The hydrated DNA (100 µM stock solution) was transferred to a 20 mM ammonium acetate solution, denatured at 95 °C for 10 min and rapidly cooled in an ice bath for 5 min. AgNO_3_ was added and the solution was incubated for 30 min at 4 °C in the dark. NaBH_4_ was added in order to reduce the silver cations and induce the AgNC formation. The ratio of the components in the final mixture was [DNA]:[Ag^+^]:[BH_4_^-^] = 1:9.6:4.8 with a final DNA concentration of 20 µM for all samples. After 24 hours incubation at 4 °C in the dark, samples were filtered 3 times by centrifugal filtration using Amicon 3kDa filters (Merck) with 20 mM ammonium acetate in order to largely remove unreacted silver or natrium borohydride residues.

**Table S1.** DNA sequences of the oligonucleotides used for the synthesis of DNA-AgNCs in this study.

| Construct Name | Sequence |  |
| --- | --- | --- |
| Ag19b-1 | 5′-TGC CTT TTG GGG ACG GAT A-3′ | [2] |
| Ag28b-1 | 5′-CAC CGC TTT TGC CTT TTG GGG ACG GAT A-3′ | [3] |

**Spectral Characteristics of the Bandpass Filters**


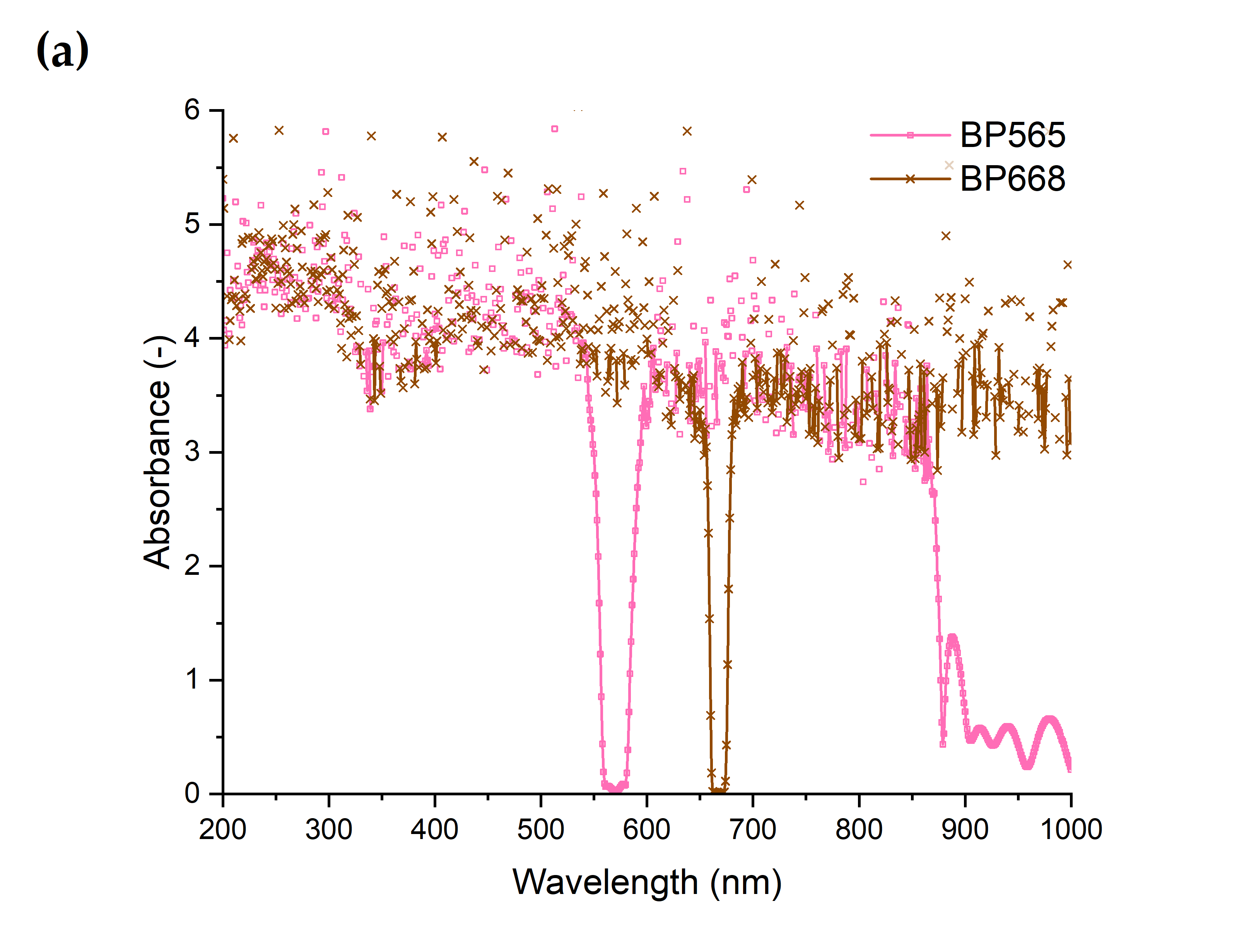

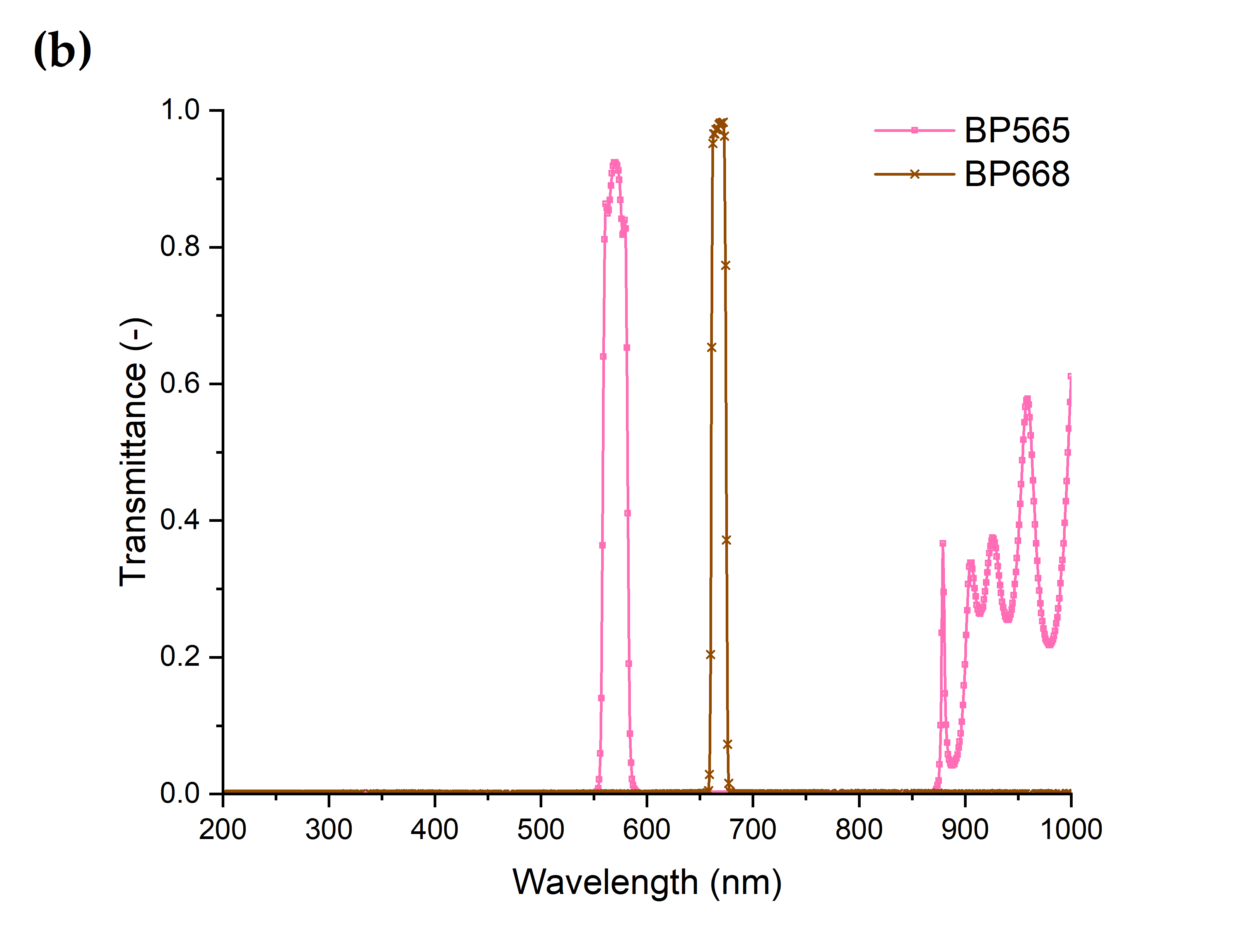


**Figure S1.** Spectra of the bandpass filters used in the setup. (a) absorbance spectrum and (b) transmission spectrum.

**Flow Rates for the Serial Dilution Experiment**

**Table S2.** Flow rates, corresponding concentrations and condition number corresponding to Figure 3 and Figure S2.

| Condition Number | [DNA-AgNC]  (nM) | 5 µM DNA-AgNC (µl/min) | 500 nM DNA-AgNC (µl/min) | 20 mM NH4OAc (µl/min) | PDMS  total (1+2)  (µl/min) |
| --- | --- | --- | --- | --- | --- |
| 1 | - | 13.3 | 13.3 | 13.3 | 30 |
| 2 | 5000 | 40 | 0 | 0 | 30 |
| 3 | 4000 | 32 | 0 | 8 | 30 |
| 4 | 3000 | 24 | 0 | 16 | 30 |
| 5 | 2500 | 20 | 0 | 20 | 30 |
| 6 | 2000 | 16 | 0 | 24 | 30 |
| 7 | 1500 | 12 | 0 | 28 | 30 |
| 8 | 1000 | 8 | 0 | 32 | 30 |
| 9 | 500 | 4 | 0 | 36 | 30 |
| 10 | 500 | 3 | 10 | 27 | 30 |
| 11 | 500 | 2 | 20 | 18 | 30 |
| 12 | 500 | 1 | 30 | 9 | 30 |
| 13 | 500 | 0 | 40 | 0 | 30 |
| 14 | 400 | 0 | 32 | 8 | 30 |
| 15 | 300 | 0 | 24 | 16 | 30 |
| 16 | 250 | 0 | 20 | 20 | 30 |
| 17 | 200 | 0 | 16 | 24 | 30 |
| 18 | 150 | 0 | 12 | 28 | 30 |
| 19 | 100 | 0 | 8 | 32 | 30 |
| 20 | 50 | 0 | 4 | 36 | 30 |
| 21 | 25 | 0 | 2 | 38 | 30 |
| 22 | 12.5 | 0 | 1 | 39 | 30 |
| 23 | 0 | 0 | 0 | 40 | 30 |
| 24 | - | 0 | 0 | 0 | 70 |

**Raw Data Overview and Data Processing**


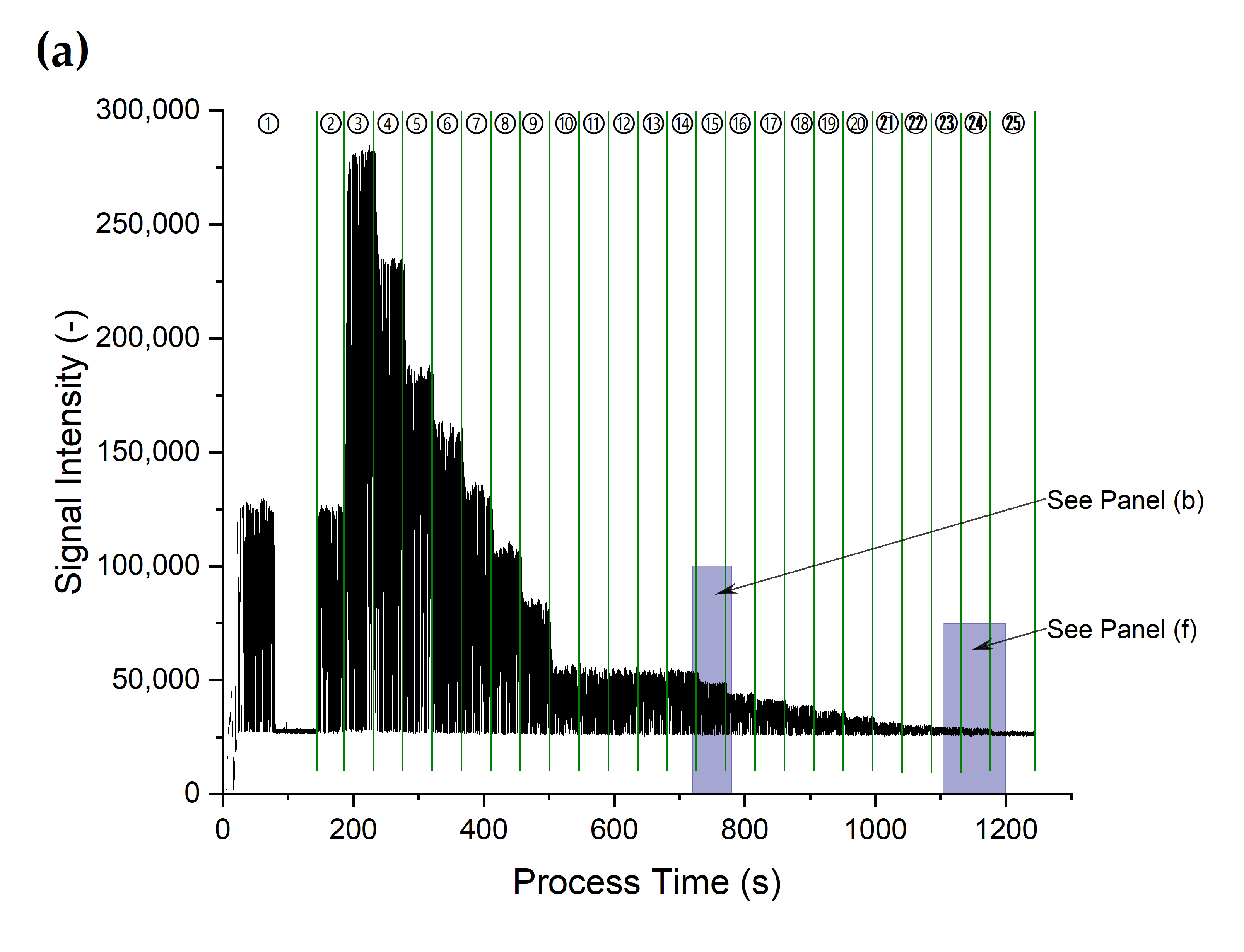

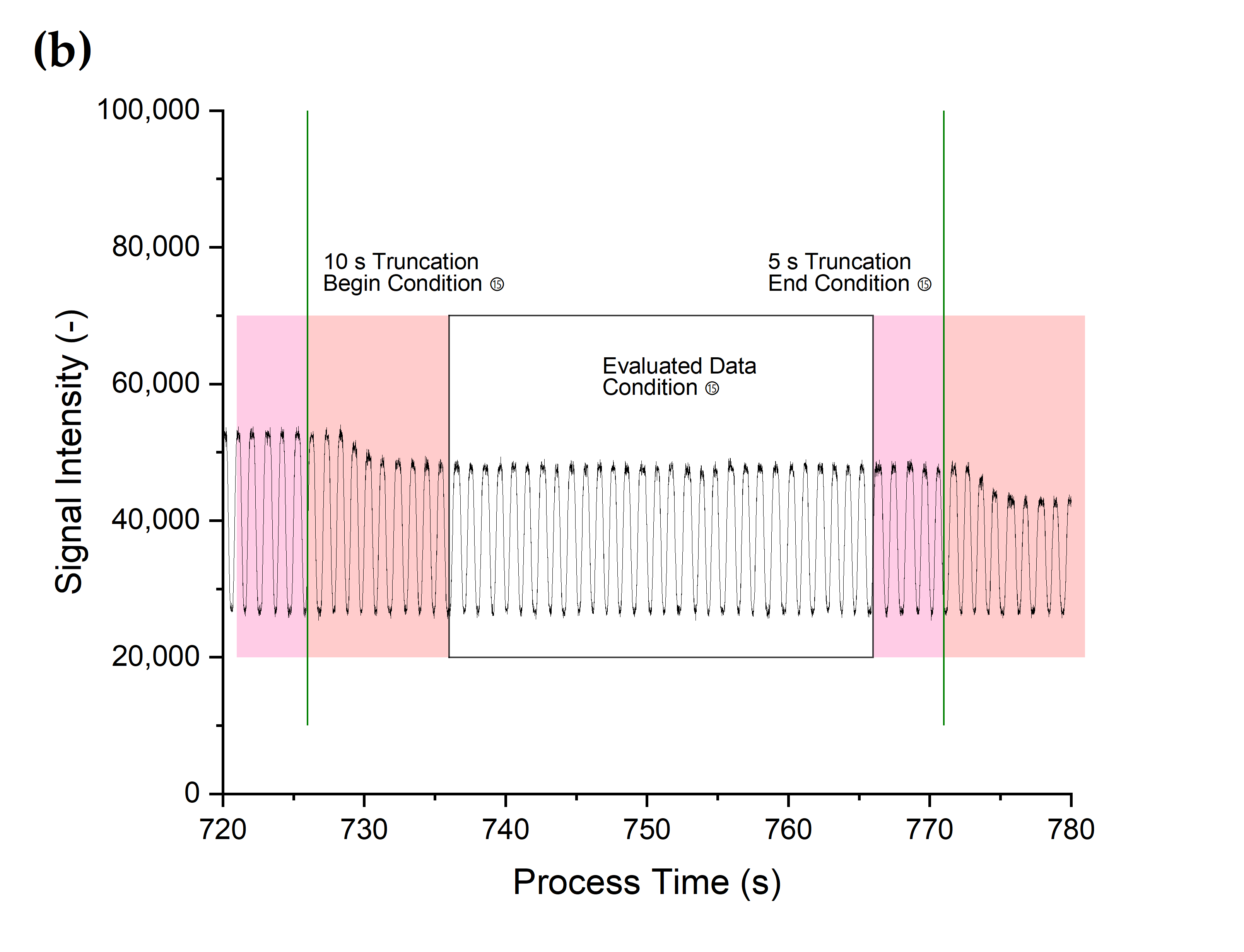

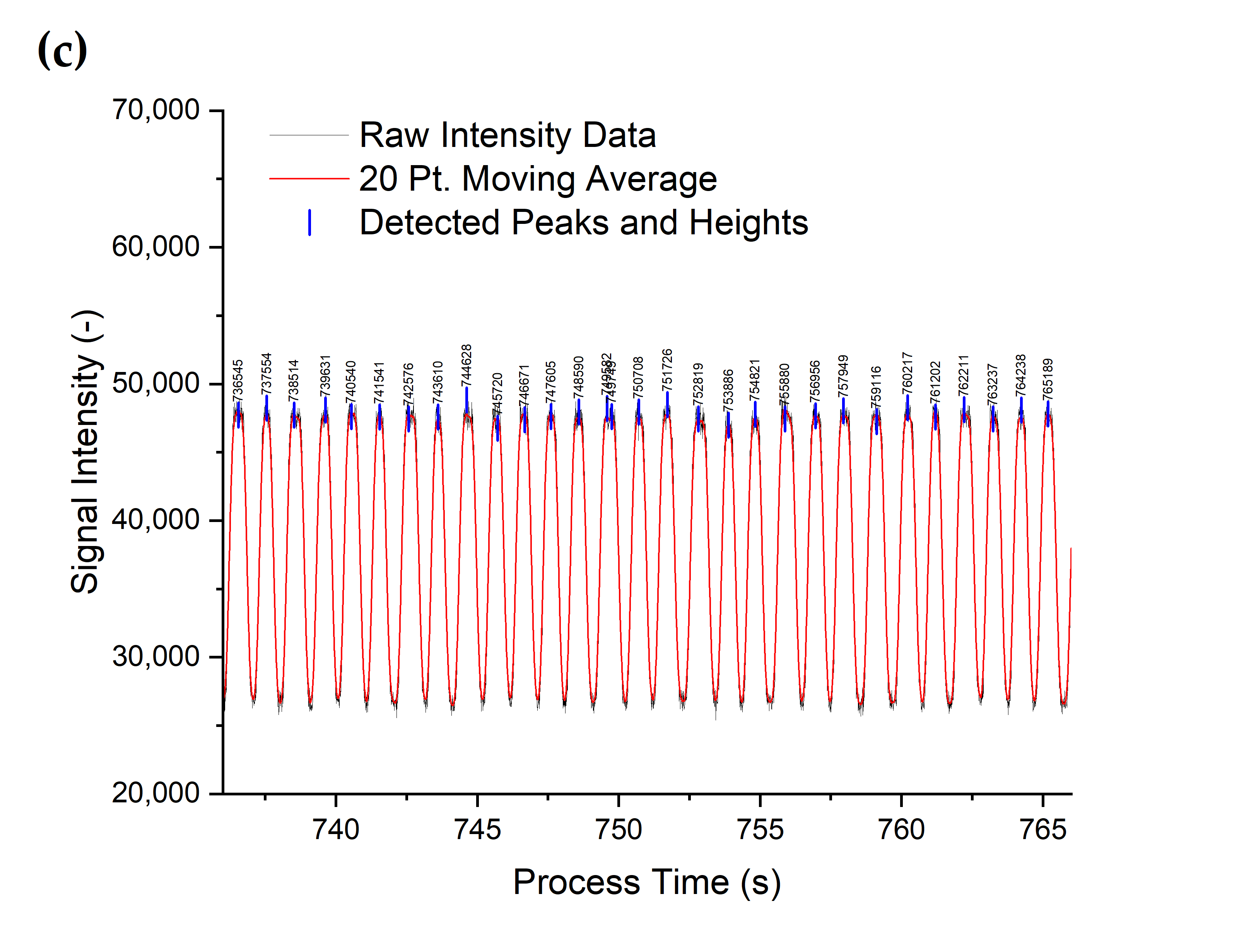

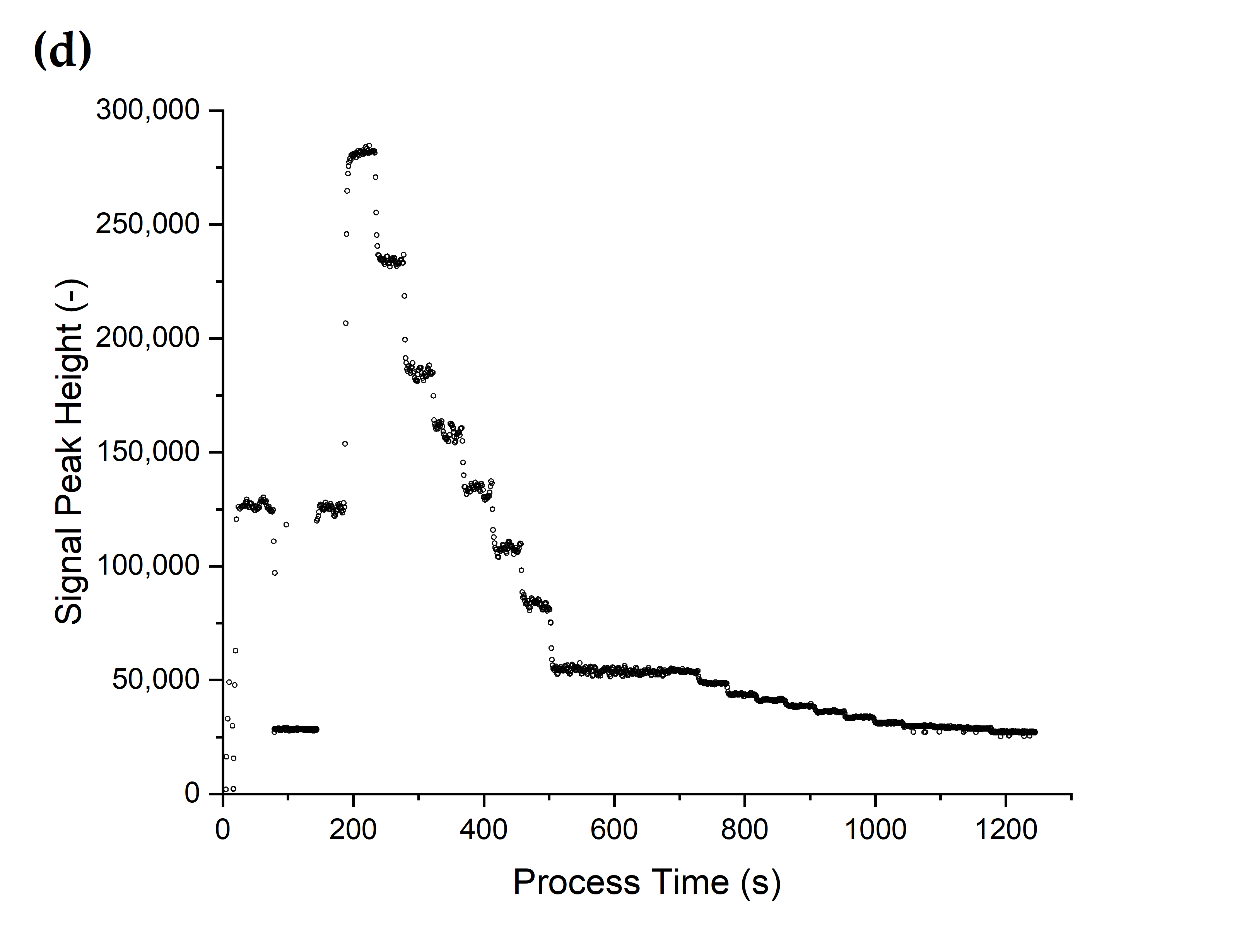

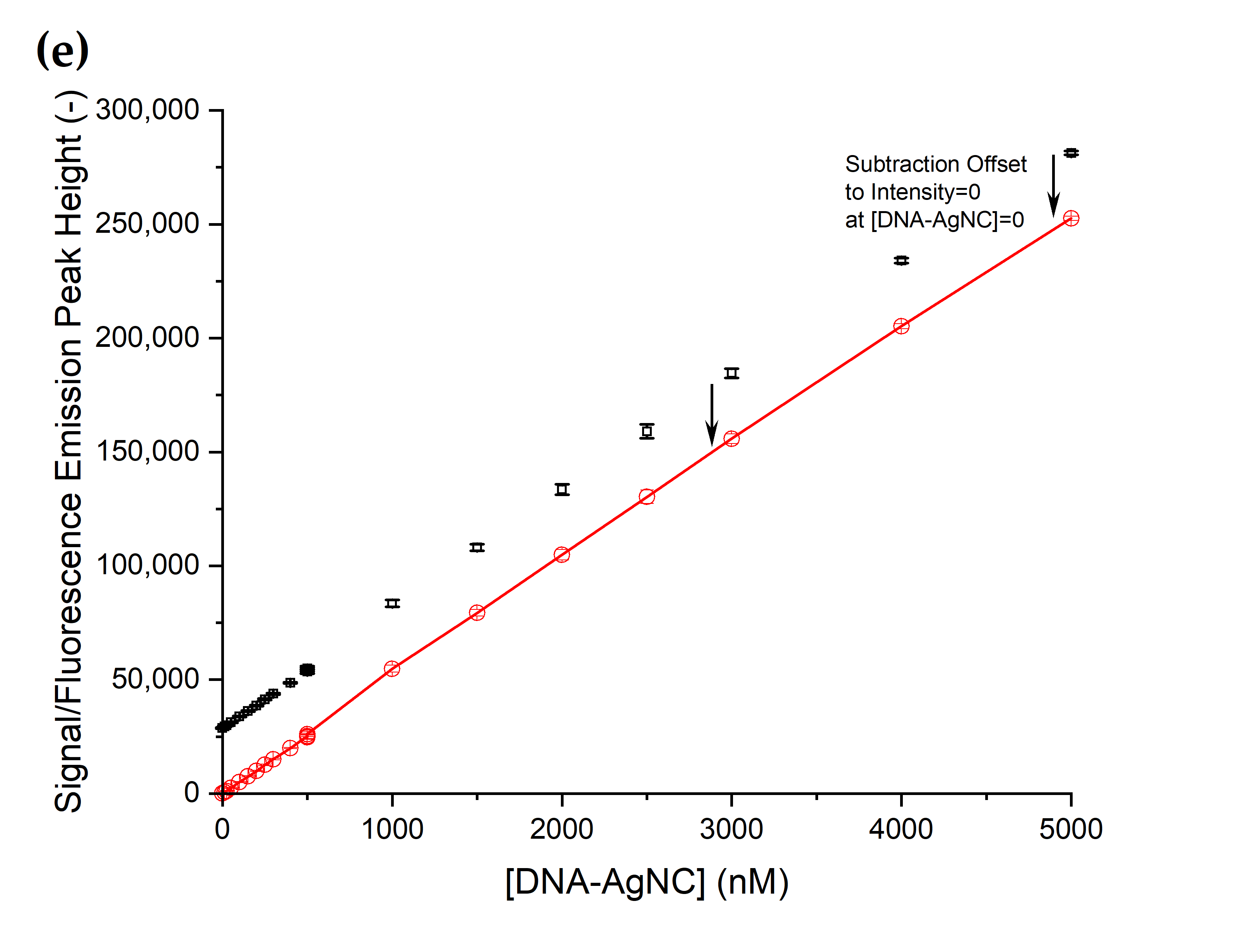

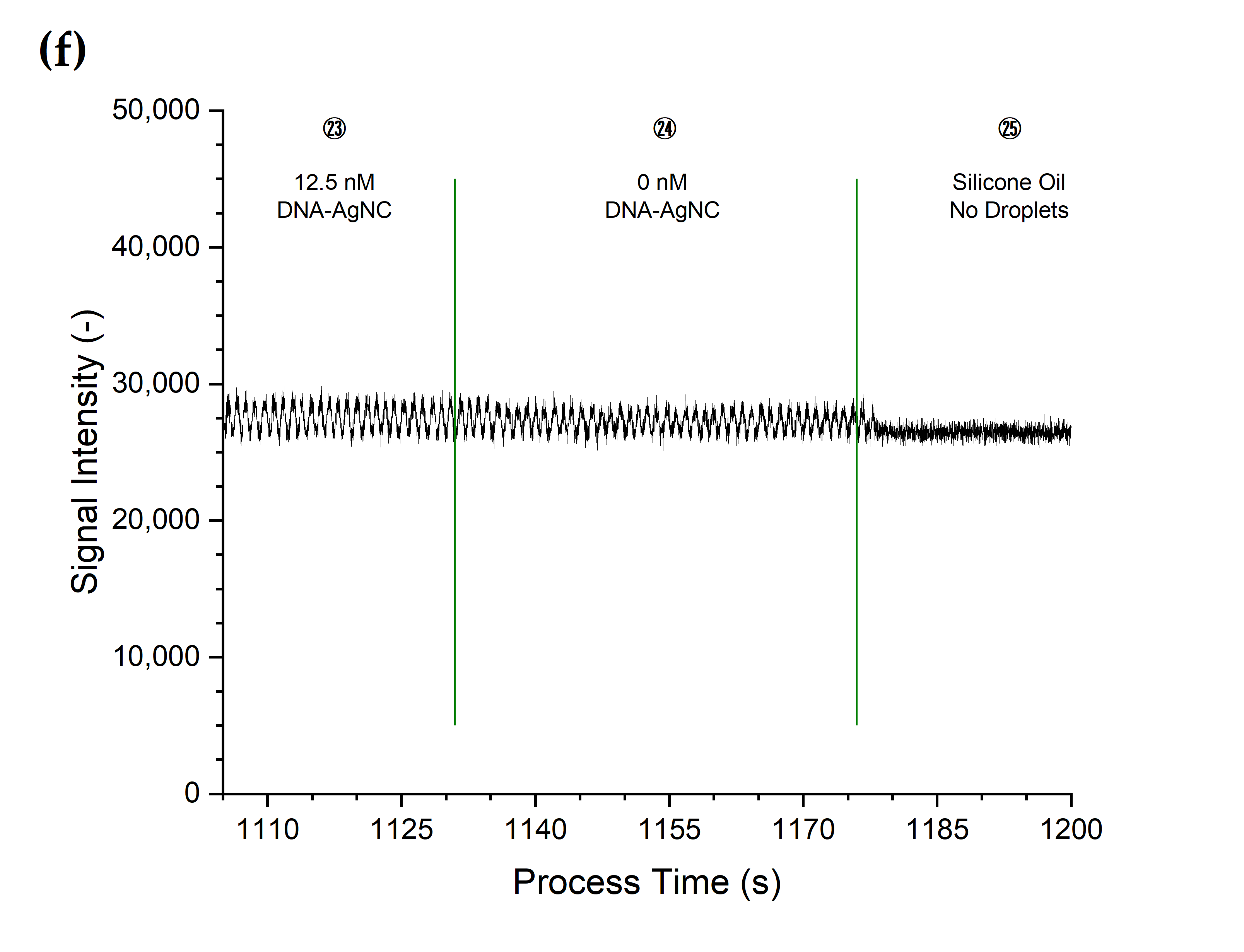


**Figure S2.** Data processing steps from the raw datatrace to the final emission intensities used for the data analysis. (a) Raw datatrace of the ADC output for channel BP668 for the dilution experiment of Ag28b1. The green, vertical lines denote changes in flow rate ratios and consequently different compositions of the droplets. (b) Detail of the raw data for condition 15, shaded areas are the truncation of the leading and trailing droplets that have been excluded from further analysis. (c) 20 pt. moving average applied to the raw data and marked detected peaks for the process time interval marked in the previous panel. (d) Plot of the height of the detected peaks over the process time. (e) Plot of the mean and standard deviation of the peak heights for every condition and offset of the data to correct for the intensity of non-fluorescing droplets. (f) Detail of the last flow conditions at 12.5 nM DNA-AgNC, 0 nM DNA-AgNC and silicone oil without droplets, respectively.

**Concentration Dependent Emission Intensity for the Construct Ag19b-1**


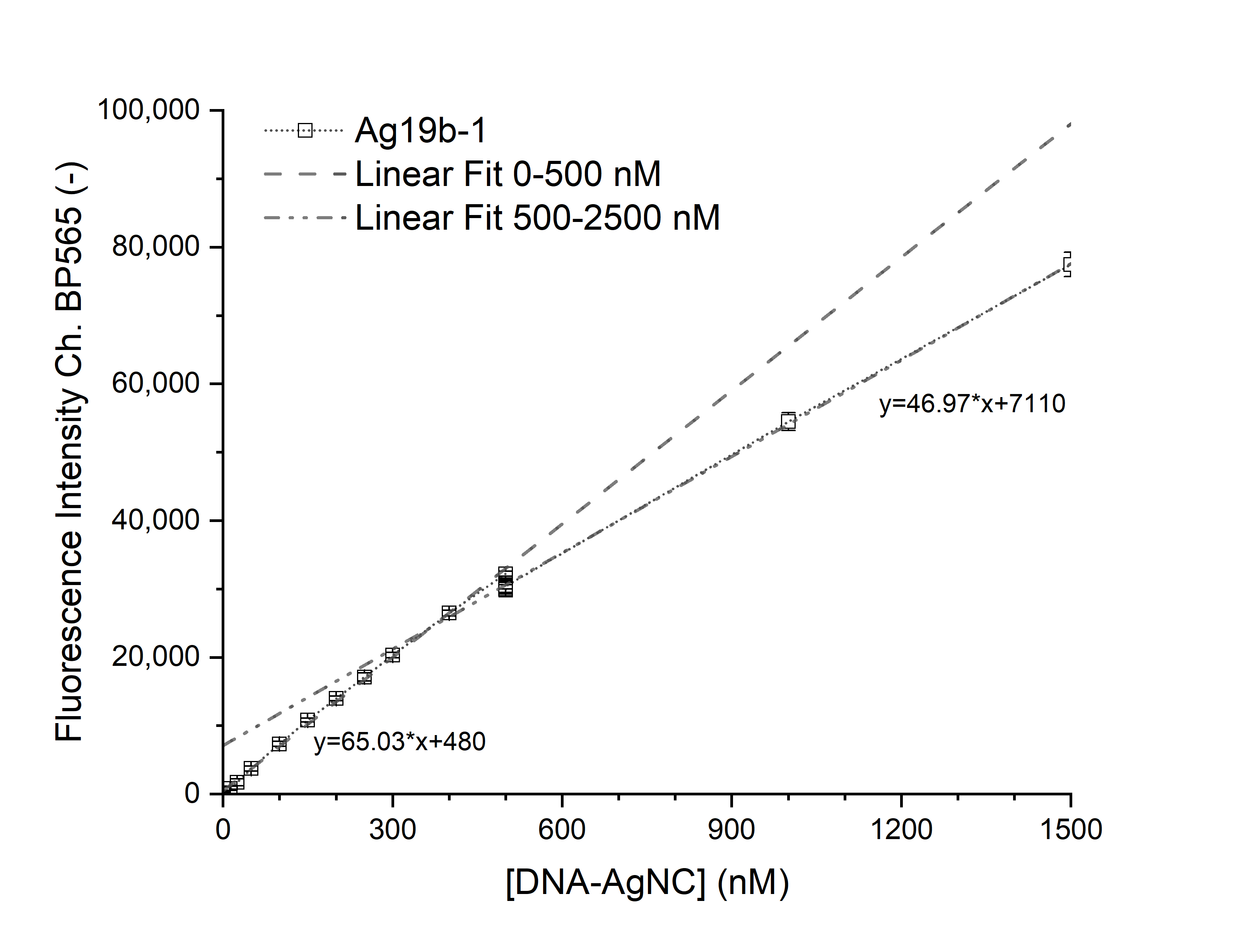


**Figure S3.** Fluorescence intensity over the concentration of Ag19b1. With linear fits for the ranges 0‑500 nM and 500‑2500 nM.

**Table S3.** Fitted parameter for the section-wise linear transfer function from concentration to fluorescence intensity for Ag19b1 for the BP668 channel.

| **Dataset** | **a** | **b** | **R²** |
| --- | --- | --- | --- |
| Ag19b1 BP565 0-500 nM | 65.03±1.0 | 480±230 | 0.9980 |
| Ag19b1 BP565 500-2500 nM | 46.97±0.19 | 7110±310 | 0.9999 |

The break in the linear relationship for Ag19b1 at 500 nM needs further investigation.

**Exploration of the Error of the NaCl-Deactivation Measurement**


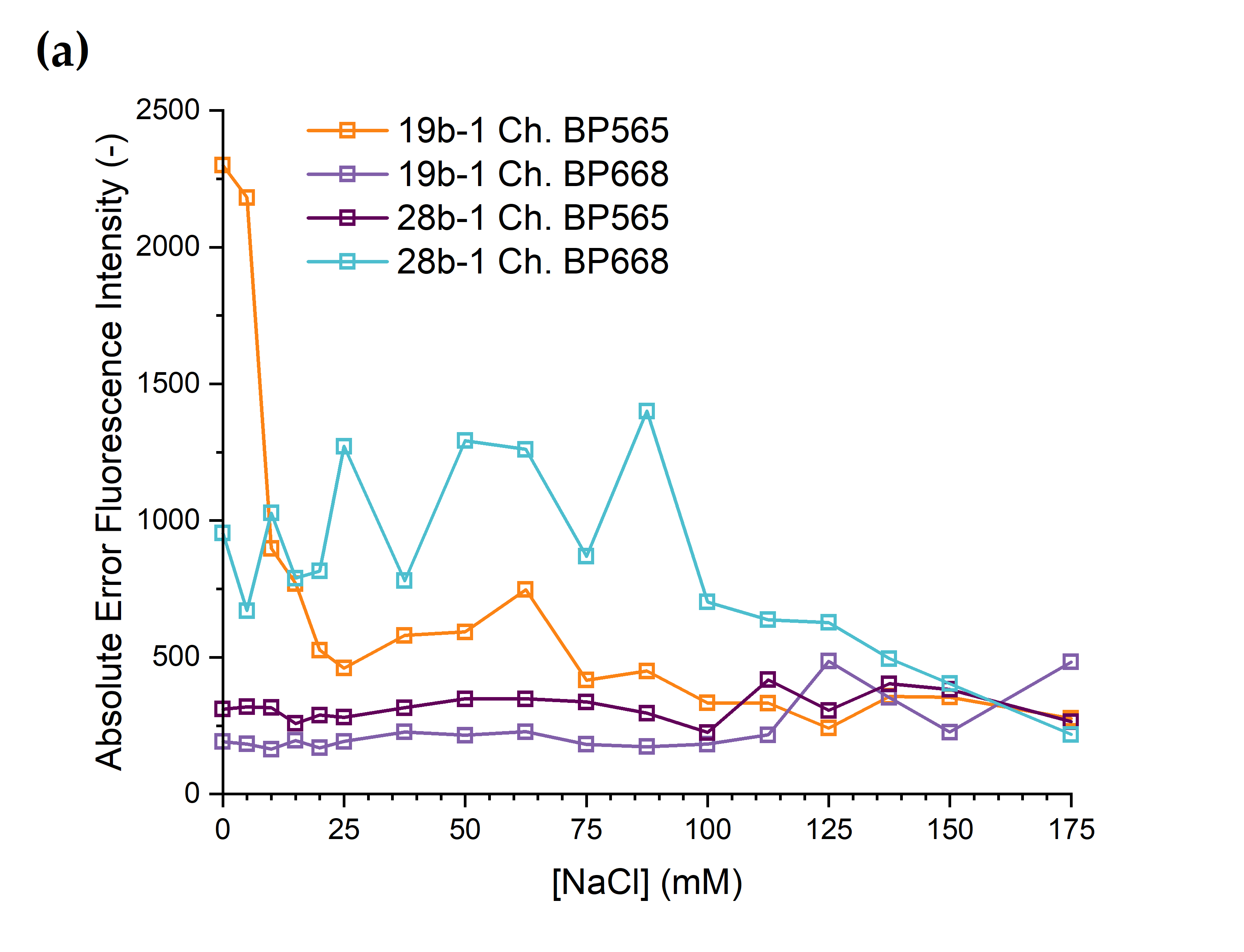

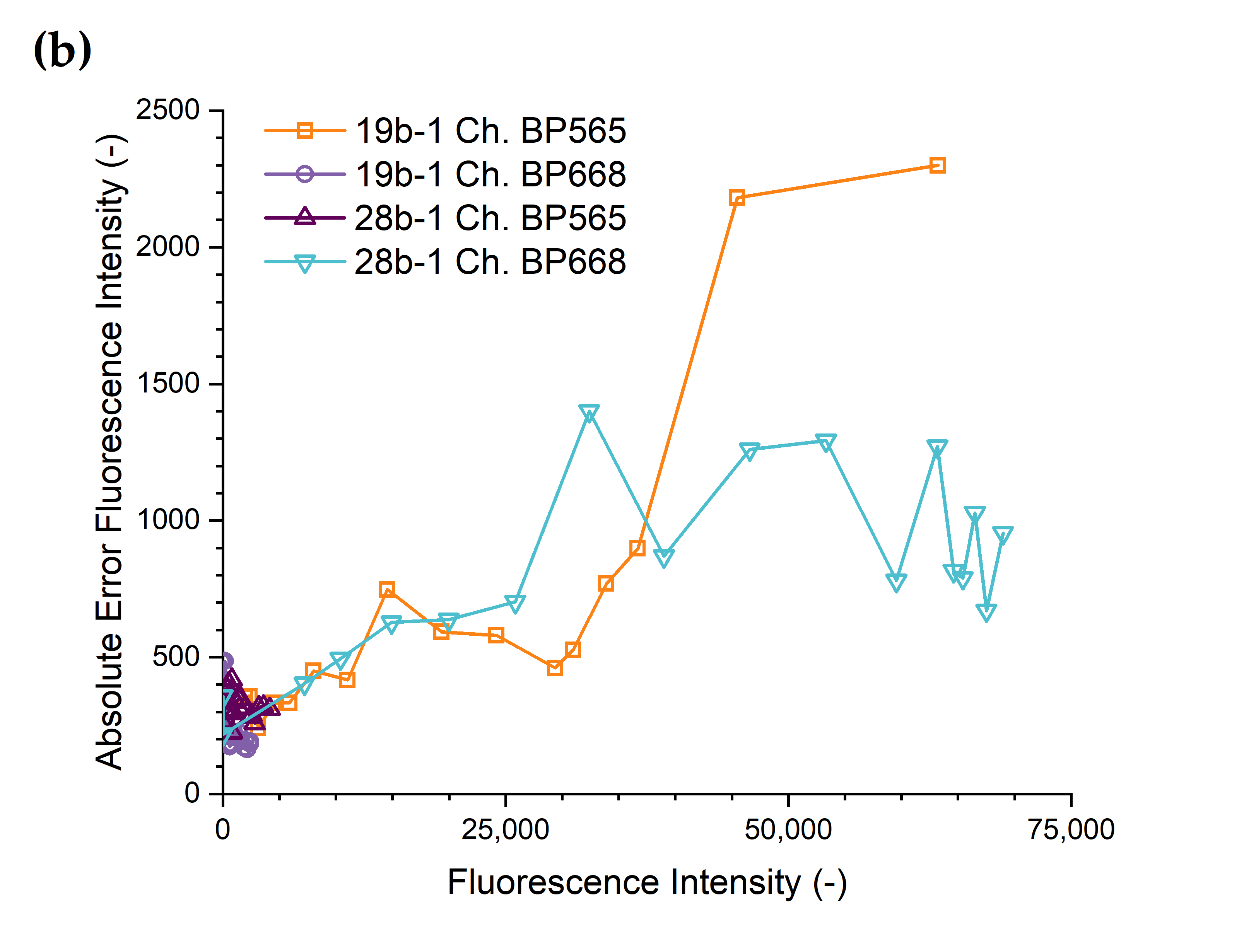


**Figure S4.** Error of the fluorescence intensity (a) over the concentration sodium chloride for all deactivation experiments and (b) over the fluorescence intensity.

References

1. Bossert, N.; De Bruin, D.; Götz, M.; Bouwmeester, D.; Heinrich, D. Fluorescence-Tunable Ag-DNA Biosensor with Tailored Cytotoxicity for Live-Cell Applications. *Sci Rep* **2016**, *6*, 37897, doi:10.1038/srep37897.

2. Schultz, D.; Gwinn, E.G. Silver Atom and Strand Numbers in Fluorescent and Dark Ag:DNAs. *Chem. Commun.* **2012**, *48*, 5748, doi:10.1039/c2cc17675k.

3. Schultz, D.; Gardner, K.; Oemrawsingh, S.S.R.; Markešević, N.; Olsson, K.; Debord, M.; Bouwmeester, D.; Gwinn, E. Evidence for Rod‐Shaped DNA‐Stabilized Silver Nanocluster Emitters. *Advanced Materials* **2013**, *25*, 2797–2803, doi:10.1002/adma.201204624.
